# Tight versus standard blood pressure control on the incidence of myocardial infarction and stroke: an observational retrospective cohort study in the general ambulatory setting

**DOI:** 10.1186/s12875-020-01163-4

**Published:** 2020-05-16

**Authors:** Bumsoo Park, Katarzyna Budzynska, Nada Almasri, Sumaiya Islam, Fanar Alyas, Rachel L. Carolan, Benjamin E. Abraham, Pamela A. Castro-Camero, Maria E. Shreve, Della A. Rees, Lois Lamerato

**Affiliations:** 1 Departments of Family Medicine and Urology, University of Michigan Medical School, Ann Arbor, MI USA; 2 Department of Family Medicine, Henry Ford Health System, Wayne State University School of Medicine, 3370 E. Jefferson Ave. Detroit, Detroit, MI 48207-4236 USA; 3 Department of Family Medicine, ProMedica Physicians Family Medicine – Monroe, Monroe, MI USA; 4 Department of Public Health Sciences, Henry Ford Health System, Wayne State University School of Medicine, Detroit, MI USA

**Keywords:** Blood pressure, Myocardial infarction, Stroke

## Abstract

**Background:**

The 2017 American College of Cardiology and American Heart Association guideline defined hypertension as blood pressure (BP) ≥ 130/80 mmHg compared to the traditional definition of ≥140/90 mmHg. This change raised much controversy. We conducted this study to compare the impact of tight (TBPC) versus standard BP control (SBPC) on the incidence of myocardial infarction (MI) and stroke.

**Methods:**

We retrospectively identified all hypertensive patients in an ambulatory setting based on the diagnostic code for 1 year at our institution who were classified by the range of BP across 3 years into 2 groups of TBPC (< 130 mmHg) and SBPC (130–139 mmHg). We compared the incidence of new MI and stroke between the 2 groups across a 2-year follow-up. Multivariate analysis was done to identify independent predictors for the incidence of new MI and stroke.

**Results:**

Of 5640 study patients, the TBPC group showed significantly less incidence of stroke compared to the SBPC group (1.5% vs. 2.7%, *P* < 0.010). No differences were found in MI incidence between the 2 groups (0.6% vs. 0.8%, *P* = 0.476). Multivariate analysis showed that increased age independently increased the incidence of both MI (OR 1.518, 95% CI 1.038–2.219) and stroke (OR 1.876, 95% CI 1.474–2.387), and TBPC independently decreased the incidence of stroke (OR 0.583, 95% CI 0.374–0.910) but not of MI.

**Conclusions:**

Our observational study suggests that TBPC may be beneficial in less stroke incidence compared to SBPC but it didn’t seem to affect the incidence of MI. Our study is limited by its retrospective design with potential confounders.

## Introduction

As one of the most common chronic health conditions, hypertension affects more than 1.3 billion adults worldwide [1] and about 75 million adults in the United States [2]. Hypertension can affect major body organs such as heart, brain, kidneys, and eyes and can lead to serious complications such as myocardial infarction (MI), stroke, end-stage renal disease, or visual impairment.

For almost three decades hypertension was defined as systolic blood pressure (SBP) ≥ 140 mmHg or diastolic blood pressure (DBP) ≥ 90 mmHg [3]. Recently, the American College of Cardiology (ACC) and American Heart Association (AHA) published a new guideline defining hypertension as blood pressure (BP) ≥ 130/80 mmHg, and set the BP treatment goal as < 130/80 mmHg [4]. This new guideline was based on the Systolic Blood Pressure Intervention Trial (SPRINT), which in 2015 showed lower rates of fatal and nonfatal major cardiovascular events and all-cause mortality when targeting SBP < 120 mmHg [5]. There have been clinical trials and meta-analyses showing that intensive blood pressure control significantly lowers the stroke risk [6–8]. Regarding the association of MI and BP control, there have been mixed results. A meta-analysis by Reboldi et al. showed no benefit in the incidence of MI compared to stroke [8]. The ONTARGET trial by Mancia et al. also found no significant benefit of intensive BP control on the MI outcome [9]. On the other hand, there have been studies that showed tight BP control might potentially increase the risk of MI [10, 11].

The new ACC/AHA guideline has raised controversies and the American Academy of Family Physicians recently decided to not endorse it due to the lack of sufficient data in systematic reviews, the lack of quality assessments for the studies, and giving considerable weight on the SPRINT trial while minimizing results from other trials [12]. Because of the controversy over the benefit of tight (TBPC) versus standard BP control (SBPC), we analyzed the incidences of MI and stroke in these two patient populations at our large health system.

## Methods

### Study design and patient population

This single-center, retrospective cohort study aimed to assess the occurrence of new MI or stroke in patients with TBPC (SBP < 130 mmHg) versus SBPC (SBP 130–139 mmHg). We addressed only SBP rather than both SBP and DBP based on similar previous studies including the SPRINT trial [5].

The data were collected and analyzed in 2018. We accessed the health system’s data stores (for medical records) to identify all outpatient encounters regardless of specialties (not limited to Family Medicine department) with ICD-10 diagnostic codes for hypertension in 2013. This resulted in 233,622 encounters in 88,456 patients. From this group we then applied inclusion and exclusion criteria to identify our study patients.

**Fig. 1:**
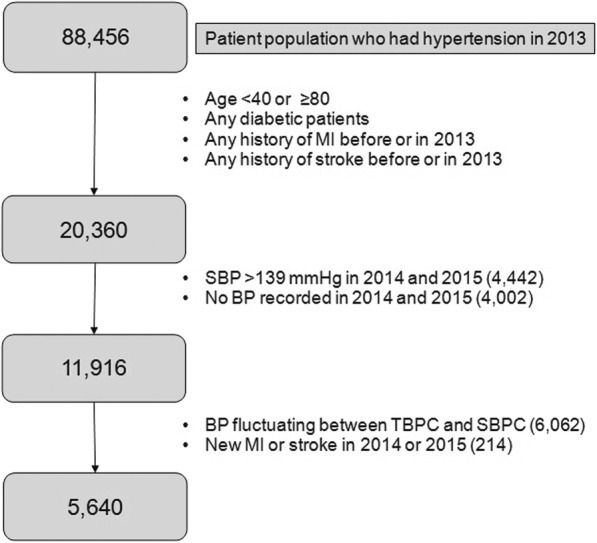
Attrition diagram of the study population

BPs have been measured by healthcare professionals manually with using a sphygmomanometer and a stethoscope in a typical outpatient setting in the same health system. We included patients with SBP < 140 mmHg between ages 40 and 79 years. The age range was based on the recent Centers for Disease Control and Prevention report showing that adults between ages 18 and 44 years mostly had hypertension under control and used antihypertensive medications less often [13]. We excluded the population greater than or equal to age 80 due to limited life expectancy. We averaged each patient’s SBPs measured in the same year between 2013 and 2015, so that one individual should have one averaged SBP reading per year. We excluded patients if the annual SBP measure was absent or if the averaged SBP fluctuated between TBPC and SBPC during the 3 years for BP characterization (2013–2015). We excluded patients with diabetes mellitus because the disease is a significant risk factor and a confounder in MI or stroke outcome. We excluded patients with a history of MI or stroke before 2013 because these are the highest risk factors for recurrent MI or stroke and we cannot assess a true incidence of new MI and stroke if we include these patients. We also excluded patients with MI or stroke events between 2013 and 2015. The attrition diagram (Fig. 1) summarizes the enrollment process.

### Outcome measures

The main outcome measures compared between the TBPC and SBPC groups were any new incidence of MI or stroke which occurred within 2 years (2016–2017). Clinical settings of the cardiovascular outcomes included outpatient, emergency department, and inpatient. For ICD-10 coding, we included MI including “late effect” codes, but we did not include angina pectoris. For stroke we counted both ischemic and hemorrhagic strokes and “late effect” codes. “Late effect” means residual sequelae after the initial acute phase of the illness has resolved [14]. We did not include transient ischemic attacks given its potential diagnostic uncertainty. We also excluded traumatic hemorrhages or vascular syndromes (e.g., vertebrobasilar artery syndrome).

### Variables

All the variables were collected through the database sorting system on Epi Info 7 provided by the Centers for Disease Control and Prevention (Atlanta, GA) per statistician (LL). We obtained baseline demographic data such as age, gender, race, and body mass index (BMI). We noted smoking status, serum low-density lipoprotein (LDL) levels, glomerular filtration rate, aspirin use, antihypertensive use, and statins use. Age was categorized by decade (40–49, 50–59, 60–69, and 70–79 years old). Race/ethnicity was categorized as African American, Asian, Hispanic, white, and other/unknown. For analysis, we further dichotomized race into 2 groups of African American and non-African American based on the recent Centers for Disease Control and Prevention report showing a relatively low percentage of hypertension visits among African Americans compared to other races [13]. BMI was categorized as underweight (BMI < 18.5 kg/m^2^), normal (BMI 18.5–24.9 kg/m^2^), overweight (BMI 25.0–29.9 kg/m^2^), obese (BMI 30.0–39.9 kg/m^2^), and morbidly obese (BMI ≥ 40.0 kg/m^2^) based on World Health Organization criteria [15]. Smoking status was categorized as “never” or “ever.” Serum LDL was categorized as ≥190 mg/dl or < 190 mg/dl; the former is one of the absolute indications of statin use based on 2013 ACC/AHA guidelines [16]. Glomerular filtration rate was categorized as ≥30 ml/min/1.73 m^2^ and < 30 ml/min/1.73 m^2^; the latter is the cutoff value where primary care physicians are recommended to refer patients to nephrology [17]. Use of aspirin, statins, and antihypertensives was determined from medication orders and defined as yes or no. In terms of counting antihypertensives, authors sorted it into major category of antihypertensives, for example, thiazide diuretic or calcium channel blockers, and then counted each medication that falls into each category of the antihypertensives per each patient during the enrollment period. This sorting process was done by statistician (LL).

### Adverse events

Authors believed it is important to evaluate hypotension-related adverse events in the TBPC group. However, given the retrospective nature of the study with using a large cohort, we were unable to collect data on the adverse events such as lightheadedness, dizziness, syncope, or falls in the TBPC group. However, we addressed and counted SBP < 90 mmHg in the TBPC group to find out the incidence of significant hypotension.

### Statistical analysis

Sample characteristics were described using means and standard deviations for continuous variables (SBP) and frequencies (number and percentage) for categorical variables (gender, race, smoking, BMI categories, use of statin, and use of aspirin). To compare the baseline variables between TBPC and SBPC groups, we conducted chi-square tests. For multivariate analysis to determine independent predictors for MI or stroke incidence, we performed binary logistic regression entering only those variables that showed significant difference between TBPC and SBPC groups with the only exception of the variable ‘antihypertensive use versus no use’ as this variable was considered to be relevant for the outcomes because antihypertensive use can modify the cardiovascular outcomes. We additionally included ‘the number of antihypertensives’ to build a different regression model to predict outcomes with excluding ‘antihypertensive use versus no use’. All statistical analyses were performed using Epi Info 7 provided by the Centers for Disease Control and Prevention (Atlanta, GA). A *P* value < 0.05 was considered statistically significant.

## Results

### Baseline data

We identified 5640 patients as a final cohort for our study (Fig. 1). Significant differences were noted in age, race, BMI, statin use, and the number of antihypertensives between the 2 groups (Table 1). More patients aged 50 to 69 years were in the TBPC group whereas the SBPC group had significantly more patients between 70 and 79 years (*P* < 0.001). African Americans were less likely to have TBPC compared to other races (*P* < 0.001). The TBPC group had more normal weight and overweight patients whereas the SBPC group had significantly more obese and morbidly obese patients (*P* < 0.001). The TBPC group had higher statin use than the SBPC group (*P* < 0.05). More patients in the SBPC group used 3 or more antihypertensive medications than those in the TBPC group. No differences were noted in gender, smoking status, serum LDL, glomerular filtration rate, and aspirin use between the 2 groups.

**Table 1:** Baseline data for patients with tight versus standard blood pressure control

Variables	TBPC (SBP < 130 mmHg) (*n* = 4530) N (%)	SBPC (SBP 130–139 mmHg) (*n* = 1110) N (%)	*P* value^a^
**Age category**			**< 0.001**
**40 to 49 years**	**778 (17.2)**	**183 (16.5)**	
**50 to 59 years**	**1533 (33.8)**	**330 (29.7)**	
**60 to 69 years**	**1466 (32.4)**	**339 (30.5)**	
**70 to 79 years**	**753 (16.6)**	**258 (23.2)**	
Gender			0.504
Male	1917 (42.3)	482 (43.4)	
Female	2613 (57.7)	628 (56.6)	
**Race**			**< 0.001**
**African American**	**1235 (27.3)**	**406 (36.6)**	
**Asian**	**152 (3.4)**	**26 (2.3)**	
**Hispanic**	**58 (1.3)**	**13 (1.2)**	
**White**	**2588 (57.1)**	**550 (49.5)**	
**Other/unknown**	**497 (11.1)**	**115 (10.4)**	
**BMI category**			**< 0.001**
**Underweight**	**50 (1.1)**	**10 (0.9)**	
**Normal**	**805 (18.4)**	**149 (13.8)**	
**Overweight**	**1602 (36.6)**	**341 (31.7)**	
**Obese**	**1624 (37.1)**	**467 (43.4)**	
**Morbidly obese**	**293 (6.7)**	**109 (10.1)**	
Smoking			0.399
Ever	2195 (48.5)	521 (47.1)	
Never	2333 (51.5)	586 (52.9)	
Serum LDL level			0.182
≥ 190 mg/dl	22 (0.6)	9 (1.0)	
< 190 mg/dl	3652 (99.4)	894 (99.0)	
EGFR (race-adjusted)			0.333
≥ 30 ml/min/1.73m^2^	3653 (99.3)	905 (99.6)	
< 30 ml/min/1.73m^2^	27 (0.7)	4 (0.4)	
Aspirin use			0.529
Yes	1073 (23.7)	253 (22.8)	
No	3457 (76.3)	857 (77.2)	
**Statin use**			**< 0.05**
**Yes**	**2008 (44.3)**	**450 (40.5)**	
**No**	**2522 (55.7)**	**660 (59.5)**	
Antihypertensive use			0.646
Yes	4328 (95.5)	1064 (95.9)	
No	202 (4.5)	46 (4.1)	
**No. Antihypertensives**			**< 0.001**
**0**	**202 (4.5)**	**46 (4.1)**	
**1**	**1711 (37.8)**	**347 (31.3)**	
**2**	**1376 (30.4)**	**321 (28.9)**	
**3 or more**	**1241 (27.4)**	**396 (35.7)**	

^a^Chi-square test

Boldface indicates statistical significance (p < 0.05)

### Incidence outcomes

The TBPC and SBPC groups showed no difference in incidence of new MI in the 2-year follow-up (Table 2). The SBPC group had a significantly higher incidence of stroke compared to the TBPC group (2.7% vs. 1.5%, *P* < 0.010) (Table 2). This gave an absolute risk reduction of 1.2%, and the number needed to treat of approximately 83.

**Table 2:** Two-year cardiovascular incidence in tight versus standard blood pressure control

Variables	TBPC (SBP < 130 mmHg) (*n* = 4530) N (%)	SBPC (SBP 130–139 mmHG) (*n* = 1110) N (%)	*P* value^a^
Myocardial infarction			0.476
Yes	28 (0.6)	9 (0.8)	
No	4502 (99.4)	1101 (99.2)	
**Stroke**			**< 0.010**
**Yes**	**68 (1.5)**	**30 (2.7)**	
**No**	**4462 (98.5)**	**1080 (97.3)**	

^a^Chi-square test

Boldface indicates statistical significance (p < 0.05)

### Multivariate analysis

In the logistic regression model, only increased age was an independent predictor of MI incidence (OR 1.518, 95% CI 1.038–2.219) when ‘antihypertensive use versus no use’ was included as one of the variables (Table 3). However, when ‘the number of antihypertensives’ was included in the model, increased number of antihypertensives was an independent predictor of MI (OR 2.272, 95% CI 1.441–3.581) (Table 4). In terms of stroke incidence, both increased age (OR 1.876, 95% CI 1.474–2.387) and TBPC (OR 0.583, 95% CI 0.374–0.910) were significantly associated with stroke incidence when ‘antihypertensive use versus no use’ was included as one of the variables (Table 3). When ‘the number of antihypertensives’ was included in the model, increased age (OR 1.792, 95% CI 1.406–2.284), increased number of antihypertensives (OR 1.282, 95% CI 1.011–1.627), and TBPC (OR 0.589, 95% CI 0.377–0.919) were independent predictors of stroke (Table 4).

**Table 3:** Multivariate analysis to predict myocardial infarction and stroke

Variables	OR (95% CI)	*P* value^a^
Myocardial infarction		
**Age**	**1.518 (1.038–2.219)**	**0.032**
African American (vs. other races)	1.010 (0.464–2.199)	0.981
Body mass index	0.828 (0.562–1.219)	0.338
Statin use (vs. no use)	1.408 (0.701–2.829)	0.314
Antihypertensive use (vs. no use)	140,232.7426 (0.000- > 1.0E12)	0.969
Tight (vs. standard) BP control	0.734 (0.339–1.588)	0.432
Stroke		
**Age**	**1.876 (1.474–2.387)**	**< 0.001**
African American (vs. other races)	1.080 (0.677–1.722)	0.748
Body mass index	0.940 (0.742–1.192)	0.610
Statin use (vs. no use)	1.338 (0.876–2.044)	0.178
Antihypertensive use (vs. no use)	1.911 (0.466–7.838)	0.369
**Tight (vs. standard) BP control**	**0.583 (0.374–0.910)**	**0.018**

^a^Binary logistic regression test

Boldface indicates statistical significance (p < 0.05)

**Table 4:** Multivariate analysis to predict myocardial infarction and stroke (Number of antihypertensives as one of the variables)

Variables	OR (95% CI)	*P* value^a^
Myocardial infarction		
Age	1.358 (0.929–1.986)	0.115
African American (vs. other races)	0.910 (0.416–1.991)	0.814
Body mass index	0.960 (0.904–1.019)	0.176
Statin use (vs. no use)	1.269 (0.630–2.557)	0.504
**Number of antihypertensives**	**2.272 (1.441–3.581)**	**< 0.001**
Tight (vs. standard) BP control	0.788 (0.363–1.707)	0.545
Stroke		
**Age**	**1.792 (1.406–2.284)**	**< 0.001**
African American (vs. other races)	1.059 (0.663–1.690)	0.812
Body mass index	0.979 (0.944–1.015)	0.243
Statin use (vs. no use)	1.300 (0.849–1.990)	0.227
**Number of antihypertensives**	**1.282 (1.011–1.627)**	**0.040**
**Tight (vs. standard) BP control**	**0.589 (0.377–0.919)**	**0.020**

^a^Binary logistic regression test

Boldface indicates statistical significance (p < 0.05)

### Adverse events

Among the 4530 patients in the TBPC group, only 5 patients (0.001%) had SBP < 90 mmHg per BP readings in the outpatient setting.

## Discussion

This single-center cohort study of more than 5000 hypertensive patients evaluated the impact of TBPC (SBP < 130 mmHg) on the incidence of new MI and stroke. We suggest that TBPC independently decreased the incidence of stroke compared to SBPC, but did not have an impact on MI incidence.

A retrospective study using the ACTION trial database found the greatest difference between SBP < 130 mmHg (2.5% incidence rate) and SBP < 140 mmHg (3.8% incidence rate) groups in the risk of stroke [18]. The cohort in the ACTION trial was different from ours in that their patients had stable coronary heart disease [19]. A meta-analysis performed by Lee et al. demonstrated that SBP < 130 mmHg compared to SBP 130–139 mmHg showed additional protective effect on stroke in patients with cardiovascular risk factors but no previously established cardiovascular disease [7]. In another meta-analysis, Reboldi et al. found that more-tight SBP control (< 120 mmHg), compared to less-tight SBP control (< 140 mmHg), significantly reduced stroke risk by 31%, but no significant risk reduction was noted in MI [8]. While these results are similar to ours, Reboldi et al’s analysis included diabetic patients and a different BP cutoff for the 2 groups. The ONTARGET trial provided further evidence on the significant impact of BP control on stroke but not on MI [9]. The trial found that more frequent achievement of target BPs (< 130/90 mmHg or < 140/90 mmHg) resulted in significant cerebrovascular protection but not cardiac protection [9]. More recent large European meta-analyses further strengthen the evidence that more intense blood pressure control reduces cardiovascular events [6, 20].

Why TBPC decreases the risk of stroke but not MI remains unclear. Several long-term prospective population-based studies have shown that hypertension increased the relative risk of stroke to a greater degree than MI [21–24]. One possible hypothesis is different blood flow physiology between the brain and heart. The brain receives a larger fraction of cardiac output by nearly 3-fold than the heart [25]. While most of the coronary blood flow occurs during diastole, the cerebral blood flow occurs during systole [26]. Therefore, cerebral blood flow might be more sensitive to a change in SBP than coronary blood flow, and coronary blood flow might be more sensitive to a change in DBP than SBP. A recent multinational study supports this hypothesis in that results showed increased risk of MI with increased DBP whereas increased SBP did not increase the risk of MI [11].

Other studies have reported different results. Verdecchia et al. performed an open-label randomized trial and found no difference in the incidence of MI or stroke between TBPC (< 130 mmHg) and SBPC (< 140 mmHg) [27]. Their population was similar to ours in the exclusion of diabetic patients. Another large randomized study using the database of the Hypertension Optimal Treatment trial showed that TBPC (< 130 mmHg) in patients with diabetes and coronary artery disease did not improve cardiovascular outcomes compared to SBPC (< 140 mmHg) [28]. The ACCORD study conducted in 2010 also showed no difference in fatal and nonfatal cardiovascular events between TBPC (< 120 mmHg) and SBPC (< 140 mmHg) groups in diabetic patients. More importantly, the TBPC group demonstrated significantly higher rates of serious adverse events attributed to antihypertensive therapy [29]. Furthermore, a post hoc analysis using the Irbesartan Diabetic Nephropathy Trial database concluded that BP ≤ 120/85 mmHg may be associated with increased cardiovascular events [30].

Our study and the majority of cohort and prospective studies have shown that TBPC is associated with decreased risk of stroke. Nevertheless, the issue of TBPC must be addressed carefully in the clinical setting. Even though cerebral blood flow has an autoregulation mechanism, the mechanism can be lost if the mean arterial BP drops below 60 mmHg [31]. A study that targeted BP < 130/80 mmHg for diabetic patients found intensive BP control caused progressive reduction of cerebral blood flow velocity [32]. According to the meta-analysis by Thomopoulos et al., the BP-lowering treatment reduced cardiovascular events by 24% whereas it increased the risk of discontinuation attributed to adverse events by 89% [33]. Ferreira et al. recently demonstrated significantly increased rates of cardiovascular death, heart failure hospitalization, MI, and stroke when SBP decreased below 125 mmHg [11]. In our study, only 0.001% (5/4530) in the TBPC group showed SBP < 90 mmHg.

An interesting finding in our study is that statin use did not independently affect MI or stroke incidence. Based on the 2013 ACC/AHA guidelines, absolute indications for statin use include clinical atherosclerotic cardiovascular disease, type 2 diabetes mellitus, and serum LDL ≥ 190 mg/dl [16]. Perhaps statin use did not affect the MI or stroke incidence in our study because we excluded patients with diabetes and those with a history of MI or stroke. Very few patients in our study had a serum LDL ≥ 190 mg/dl (Table 1). Another interesting finding is that increased number of antihypertensive medications significantly predicted both MI and stroke incidence while the usage of antihypertensive medication itself did not predict either MI or stroke incidence in the multivariate model. This can suggest that requiring more antihypertensive medications rather than taking the medications itself could predict higher cardiovascular risk, or those who were prescribed many antihypertensive medications would have not been adherent to the medications.

### Limitations

The major limitation of our study is that this is an observational retrospective study. Given the fact that authors did not do any interventional measures for the patients, strictly speaking, it might not be very appropriate to use the terms “tight control” and “standard control”. However, as all the patients were formally diagnosed with hypertension, authors assumed that they had received some type of BP control intervention including lifestyle counseling from their providers. And this would have justified our using the terms “control.” Our study also has several other limitations. Because of the retrospective design, we were not able to completely address and remove all potential confounders. For example, excluding patients whose BP showed fluctuating levels between TBPC and SBPC could otherwise have affected the outcome. There were significant differences in age, race, BMI, statin use, and the number of antihypertensives between TBPC and SBPC groups. The TBPC group was younger and had statins prescribed more often than the SBPC group. Although these variables were adjusted at multivariate analysis, these differences might still have affected our findings, and we admit that this is potentially the most major limitation of our study, which should be criticized. Also, as we excluded other significant risk factors of MI and stroke, such as diabetes and history of MI or stroke, our study population was different from actual patients commonly seen in clinical practice, which might have resulted in the relatively rare outcome events in our study. Also, as we were not able to review each patient’s medical record given the large, retrospective, and electronic-based data analysis, we were unable to collect information on the adverse events that can potentially occur from TBPC such as dizziness, syncope, falls, acute kidney injury, or cerebral hypo-perfusion. However, given that only 0.001% of the TBPC patients had SBP less than 90 mmHg, it is less likely that many of our TBPC patients developed significant hypotension-related complications. Also, we were unable to assess each patient’s medication adherence data as we only counted the numbers of the medication prescriptions which can potentially be significantly confounding. It also would have been more informative if we could include the mortality and quality of life outcomes in either group, which should be considered for future research.

## Conclusions

Our observational retrospective cohort study showed that TBPC had a significant benefit in less stroke incidence compared to SBPC, although it did not affect the incidence of MI. Our study could help aid in the discussion regarding the association of BP control and stroke in actual clinical practice. Even though the American Academy of Family Physicians decided to not endorse the ACC/AHA guideline, our study shows more favorable result towards the SPRINT trial in terms of less stroke incidence.

## Supplementary information

**Additional file 1.** STROBE Statement—Checklist of items that should be included in reports of observational studies.

## Data Availability

The datasets used and/or analyzed during the current study are available from the corresponding author on reasonable request.

## Abbreviations

ACC: American College of Cardiology
AHA: American Heart Association
BMI: Body mass index
BP: Blood pressure
DBP: Diastolic blood pressure
LDL: Low-density lipoprotein
MI: Myocardial infarction
SBP: Systolic blood pressure
SBPC: Standard blood pressure control
SPRINT: Systolic Blood Pressure Intervention Trial
TBPC: Tight blood pressure control
